# A novel quantification method for the total demethylation potential of aquatic sample extracts from Bohai Bay using the EGFP reporter gene

**DOI:** 10.1186/s12896-015-0224-y

**Published:** 2015-11-26

**Authors:** Yan Qian, Xiaoli Wang, Zhanlu Lv, Chen Guo, Mei Han, Jiabing Wu, Yongjian Yang, Yishu Yang, Yan Jiang, Yongjie Wei, Jing Nie, Bao Liang, Jinliang Zhang, Xianliang Wang

**Affiliations:** State Key Laboratory of Environmental Criteria and Risk Assessment, Chinese Research Academy of Environmental Sciences, Beijing, 100012 China; College of Life Science and Bioengineering, Beijing University of Technology, Beijing, 100124 China; School of Public Health, Anhui Medical University, Hefei, 230032 China; Institute of Environmental Health and Related Product Safety, Chinese Center for Disease Control and Prevention, Beijing, 100021 China

**Keywords:** Demethylation, Aquatic sample, EGFP, Metals, Bohai bay

## Abstract

**Background:**

The demethylation potential of environmental pollutants is possibly an innate part of their comprehensive health risk. This paper develops a novel method called TDQ to quantify the demethylation epigenetic toxicity, termed the 5-AZA-CdR demethylation toxic equivalency, of aquatic samples from the heavily polluted Bohai Bay using Hep G2 cell lines transiently transfected with the pEGFP-C3 plasmid containing a methylated promoter of the EGFP reporter gene inserted artificially *in vitro*.

**Results:**

If the aquatic sample extract has strong total demethylation potential to the promoter, its methylation level will decrease, and increased green fluorescence will be observed under microscopy after TDQ co-incubation. The 5-AZA-CdR was selected as a representative demethylation agent to validate the principle of the TDQ method on three levels: significant dose–response relationships between the concentration of 5-AZA-CdR and the methylation level of promoters, mRNA expression level of the EGFP gene, and the fluorescence intensity of EGFP proteins. Twenty extracts from aquatic samples are successfully quantified with the TDQ test. Eight of them return meaningful results ranging from 0.00004 to 0.20053 μM 5-AZA-CdR toxicity equivalents.

**Conclusions:**

The TDQ method is a reliable and rapid assay for the quantification of the DNA demethylation potential of aquatic sample extracts, which may shed light on the safety evaluation of food material.

**Electronic supplementary material:**

The online version of this article (doi:10.1186/s12896-015-0224-y) contains supplementary material, which is available to authorized users.

## Background

Health risks from environmental pollution in China have become increasingly important to public concern, especially in terms of food safety. Many toxicological methods have been invented to test for the presence of gene mutations and chromosome aberrations due to environmental pollutants, such as the Salmonella reverse mutation assay for mutagenic activities and the comet assay for chromosome damage [1]. Some research has shown that epigenetic negative effect of environmental pollutants in food materials cannot be overlooked. The epigenetic mechanism has become one of the focusing fields for the etiology of many disorders, including the adverse effect of many environmental pollutants, such as heavy metals, during the last decade. The primary function of DNA methylation for regulatory elements is to down-modulate gene expression. Epigenetic changes, including global DNA hypomethylation and hypermethylation of tumor suppressor genes, are frequently observed in cancer cells. DNA methylation is susceptible to change and an excellent candidate for explaining how certain environmental factors may increase the risk of cancer [2, 3].

Cadmium increases DNA methylation and the expression of DNMT1 and DNMT3a mRNA in the livers and kidneys of hens, implying that DNA methylation may be involved in the carcinogenic action of cadmium [4]. Environmental cadmium exposure was associated with DNA hypomethylation in peripheral blood in Argentinean women [5]. The genetic reactivation of long interspersed nuclear element-1 by benzo(a)pyrene involves reduced DNA methylation of CpG islands [6]. Many pollutants appear to have the potential to change the DNA methylation status of key genetic elements, which may be involved in the disorder progression from environmental pollutants in many types of food material.

According to the 2013 China Marine Environmental Quality Communique [7], the costal line water in Bohai Bay abutting the Tianjin metropolitan area has been ranked worst for pollution for many years. Even the living marine mollusks in the coastal sea area abutting the pollution sources are marked by serious heavy metal contamination [8, 9]. The total toxic demethylation potential of aquatic samples should be examined to evaluate their safety for local residents. Thus far, few studies have reported on assessment methods of DNA methylation alternation for food material. One study reports on the establishment of a detection system for demethylating agents using an endogenous promoter CpG island [10], but the HCT 116 cell line is a poor candidate tool due to its relative absence of the cytochromes P450 enzymes and corresponding activation of pollutants. Another two-component reporter gene system has been described based on the visualization and quantization of dynamic changes in targeted DNA methylation in bone marrow-derived stem cells or cancer cell lines [11].

**Fig. 1:**
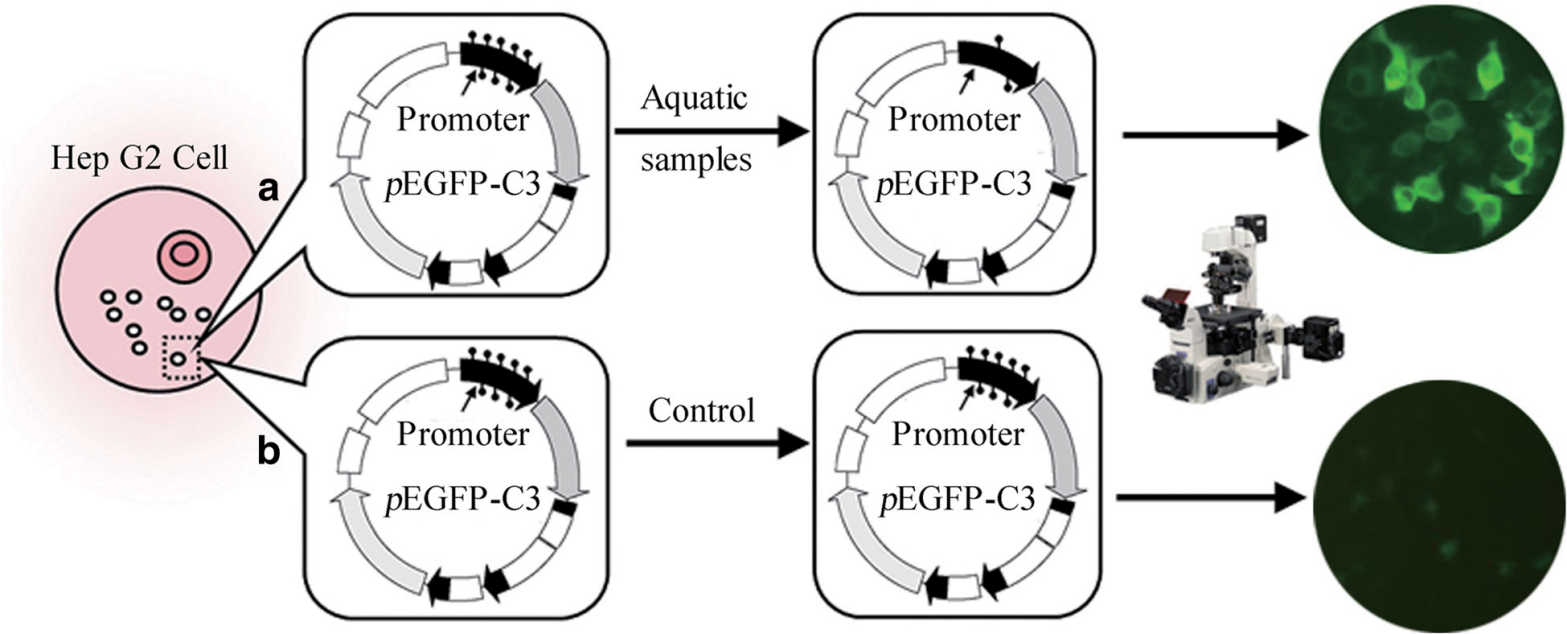
TDQ schematic of total demethylation potential quantification method for aquatic samples. **a** Aquatic samples containing pollutants of strong demethylation potential as 5-AZA-CdR. **b** Control or Aquatic samples containing pollutants of weak demethylation potential. pEGFP-C3, pEGFP-C3 plasmid vector. Promoter, the CMV promoter in pPEGFP-C3 vector. Small stick with round point end in the promoter area represents the methyl (CH_3_)

The assessment of DNA methylation status is proposed for safety assessment, and the evaluation of the overall methylation status is desirable [12–15]. In the present study, which aims to develop a demethylation toxicity assessment of aquatic samples that can quickly identify potential health risks through epigenetic mechanisms, we report a novel method for the total demethylation potential quantification (TDQ) with the EGFP reporter gene and the artificially methylated promoter in Hep G2 cell lines (Fig. 1).

## Results

### Validity of TDQ test for demethylation potential

The 5-aza-2'-deoxycytidine (5-AZA-CdR) was selected as a representative positive demethylation agent to validate the principle of the TDQ method (Fig. 1) on three levels: dose–response relationship exploration between the concentration of 5-AZA-CdR and the methylation level of CMV promoters, mRNA expression level of EGFP gene, and the intensity of cellular fluorescence intensity (Table 1). The Hep G2 cells with successful transfection of pEGFP-C3 plasmids containing methylated promoters grew well after the cell incubation conditions were optimized.

**Table 1:** The effect of 5-AZA-CdR on the EGFP reporter gene in cells with the TDQ experiment

Groups	n	5-AZA-CdR (μM)	Methylation level (%)	EGFP mRNA level	EGFP fluorescence (%)
A0	5	0.00000	91.6 ± 3.5	1.00 ± 0.00	2.20 ± 0.47
A1	5	0.00016	87.8 ± 2.1	1.65 ± 0.34	7.68 ± 0.87
A2	5	0.00080	85.5 ± 2.5	1.77 ± 0.26	8.75 ± 1.06
A3	5	0.00400	79.8 ± 3.6	1.92 ± 0.35	9.81 ± 1.29
A4	5	0.02000	74.5 ± 3.2	12.30 ± 0.93	11.18 ± 1.39

Note. Cell groups A0 ~ A4 are treated with different concentrations of 5-AZA-CdR. Methylation levels are the concrete methylation levels of the CMV promoter within Hep G2 cell groups with BSP analysis. EGFP mRNA level are the concrete relative level of EGFP mRNA within Hep G2 cell groups with real-time PCR analysis. EGFP fluorescence is the concrete intensity of fluorescence of Hep G2 cell groups with cytometry analysis. There are significant differences for ANOVA analysis of the methylation level between all groups barring groups A0 and A1and groups A1and A2. There are significant differences for ANOVA analysis of the EGFP mRNA level between all groups barring groups A1 and A2, groups A2 and A3, and groups A1 and A3. There are significant differences for ANOVA analysis of the EGFP fluorescence between all groups barring groups A1 and A2, groups A2 and A3, and groups A3 and A4

### Methylation level of CMV promoter and the treatment of 5-AZA-CdR

**Fig. 2:**
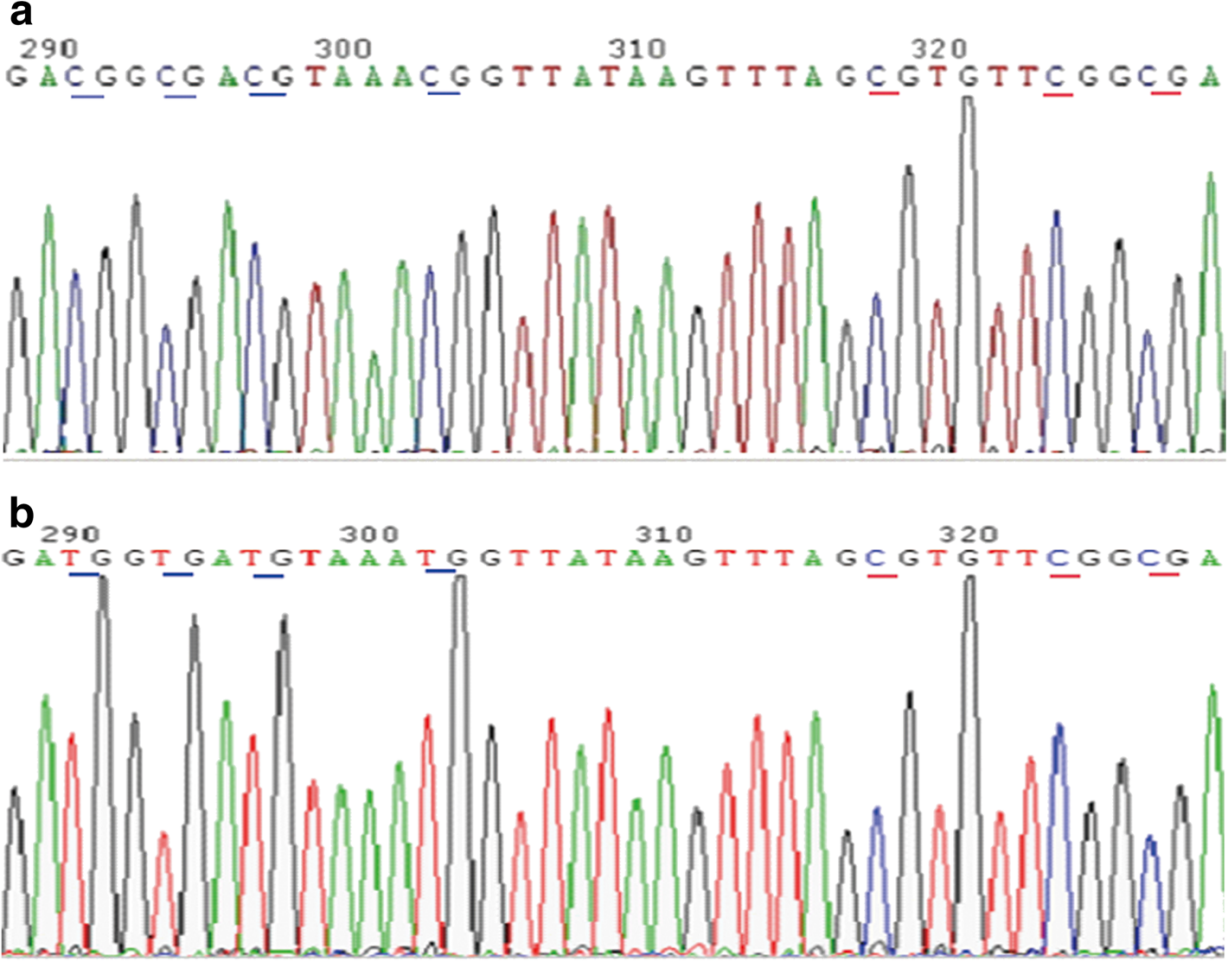
The representative sequence maps for BSP methylation quantification of the CMV promoter in cells. This map is for the 290–330 bp of the PCR T clones for sequencing. The upper map (**a**) for the control group: 7 mCGs (blue peaks) did not change after bisulfite treatment and sequencing. The lower map (**b**) for 5-AZA-CdR group: 4 mCGs changed to TGs (red peaks to the left) due to the methyl lost and 3 mCGs did not change (blue peaks to the right) after bisulfite treatment and sequencing

**Fig. 3:**
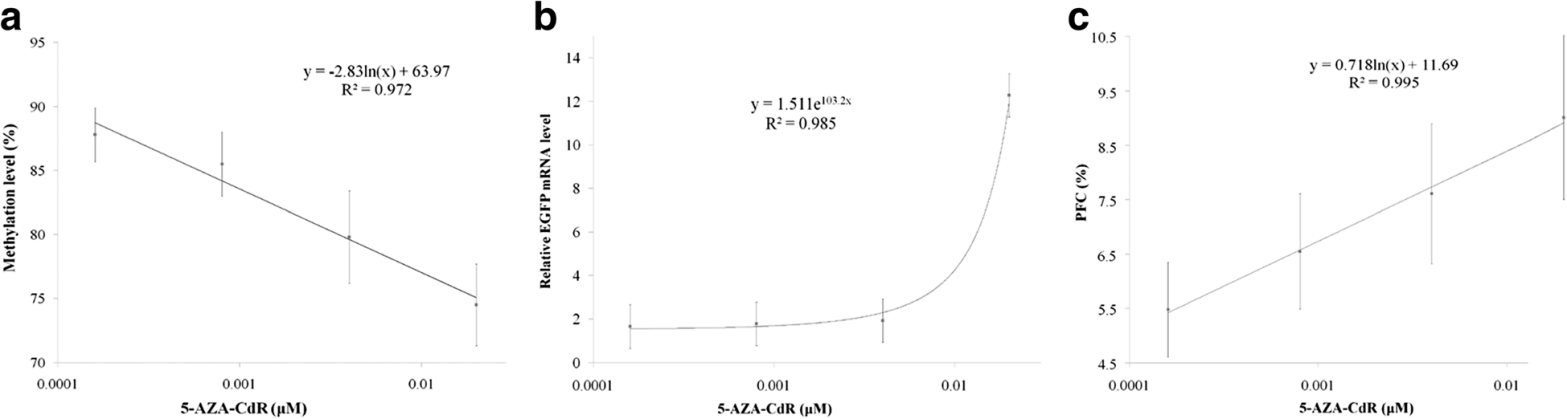
Dose-effect relationships between the 5-AZA-CdR and the methylation level, EGFP mRNA and PFC of cells. **a** The 5-AZA-CdR and the methylation level of the CMV promoter. **b** The 5-AZA-CdR and the relative folds of EGFP mRNA. **c** The 5-AZA-CdR and the percentage of positive fluorescence cells (PFC)

The bisulfite treatment of DNA converts all unmethylated cytosines to uracils, whereas methylated cytosine is unchanged under the reaction condition; thus, methylation status can be determined from different sequences [16]. The decreasing methylation levels of the CMV promoter were observed from cells treated with increasing 5-AZA-CdR doses with BSP results. The representative BSP results are shown in Fig. 2. There is a significant dose-effect relationship expressed as y = −2.83ln(x) + 63.97 (R^2^ = 0.972) between the 5-AZA-CdR treatment and the methylation level of the CMV promoter from corresponding cells in the TDQ test (Fig. 3a), which indicates that CMV promoter methylation is a valid candidate test target for potential demethylation agents.

### Expression level of the EGFP gene and the treatment of 5-AZA-CdR

To explore the response of the EGFP gene expression to the treatment of 5-AZA-CdR gradients in the TDQ experiment, the relative mRNA volume of the EGFP gene within the C3 plasmid was evaluated based on a reference GAPDH gene. Increasing expression levels of the target gene from different cell wells were found with increasing concentrations of 5-AZA-CdR (Additional file 1: Table S1). A significant dose-effect relationship between the 5-AZA-CdR treatment and the relative folds of EGFP mRNA was confirmed with an equation of y = 1.511exp(103.2x) (R^2^ = 0.985) (Fig. 3b), which indicated that the expression of EGFP mRNA within the C3 plasmid in the Hep G2 cell lines is sensitive to the treatment of 5-AZA-CdR.

### Fluorescence intensity of EGFP and treatment of 5-AZA-CdR

**Fig. 4:**
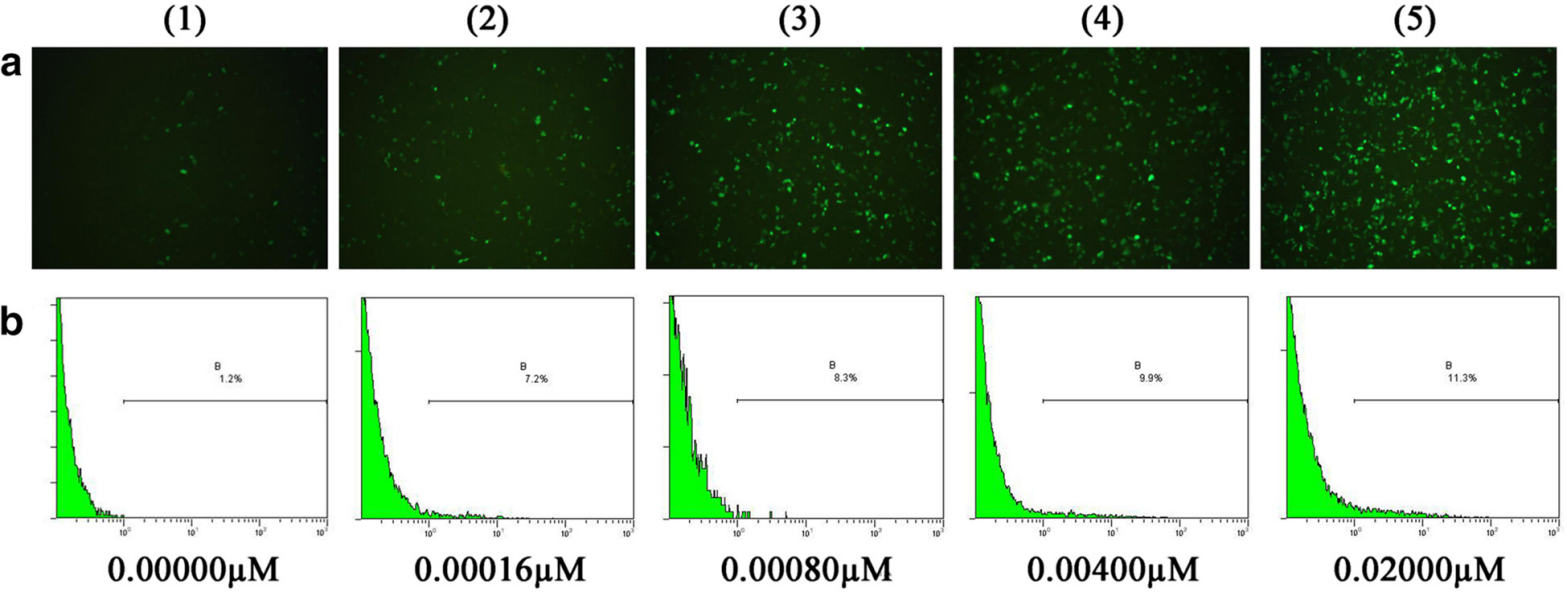
The representative images and flow cytometry results of different cells treated with 5-AZA-CdR gradients. The upper series (line **a**) are for fluorescence images (with magnification of 100-folds) and the lower series (line **b**) are for flow cytometry measurement from the control group and the 5-AZA-CdR treatment groups with increasing doses (0.00000, 0.00016, 0.00080, 0.00400, 0.02000 μM)

To explore the response of cells’ EGFP fluorescence intensity to the treatment of 5-AZA-CdR gradients in the TDQ experiment, the spontaneous fluorescence intensity of cells with increasing concentrations of 5-AZA-CdR (Table 1) was evaluated directly via microscopy imaging, and later by using flow cytometry measurement to find the percentage of positive fluorescent cells. The cells treated with higher concentration of 5-AZA-CdR have significantly brighter images than those treated with correspondingly lower concentrations (Fig. 4). A significant dose-effect relationship between the 5-AZA-CdR treatment and percentage of positive fluorescent cells was confirmed and expressed by y = 0.718ln(x) + 11.69 (R^2^ = 0.995) (Fig. 3c), which indicated that the spontaneous fluorescence of the EGFP protein from the gene located in the pEGFP-C3 plasmid in the Hep G2 cell lines was sensitive to the treatment of 5-AZA-CdR and possibly to other agents with demethylation potential.

### Detection limit of the TDQ test for 5-AZA-CdR experiment

To explore the detection limit of the TDQ assay, five samples with relatively lower concentrations of 5-AZA-CdR (0.0000025, 0.00001, 0.00004, 0.00016, 0.00064 μM) were prepared for TDQ test (Additional file 2: Table S2). Results showed that the detectable noise signals varied from 0.0000025 μM to 0.00001 μM, and meaningful fluorescence intensity of cells was observed with a concentration higher than 0.00004 μM 5-AZA-CdR. No significant difference in fluorescence intensity was found between the cell groups treated with 5-AZA-CdR (0.0000025 and 0.00001 μM) and the control cell group, while a significant difference in fluorescence intensity was observed between the cell groups treated with 5-AZA-CdR (0.00004, 0.00016 and 0.00064 μM) and the other cell groups (0, 0.0000025 and 0.00001 μM). Therefore, we recognized that the detection limit of TDQ is approximately 0.00004 μM 5-AZA-CdR.

### Accuracy and precision of the TDQ test for 5-AZA-CdR experiment

**Fig. 5:**
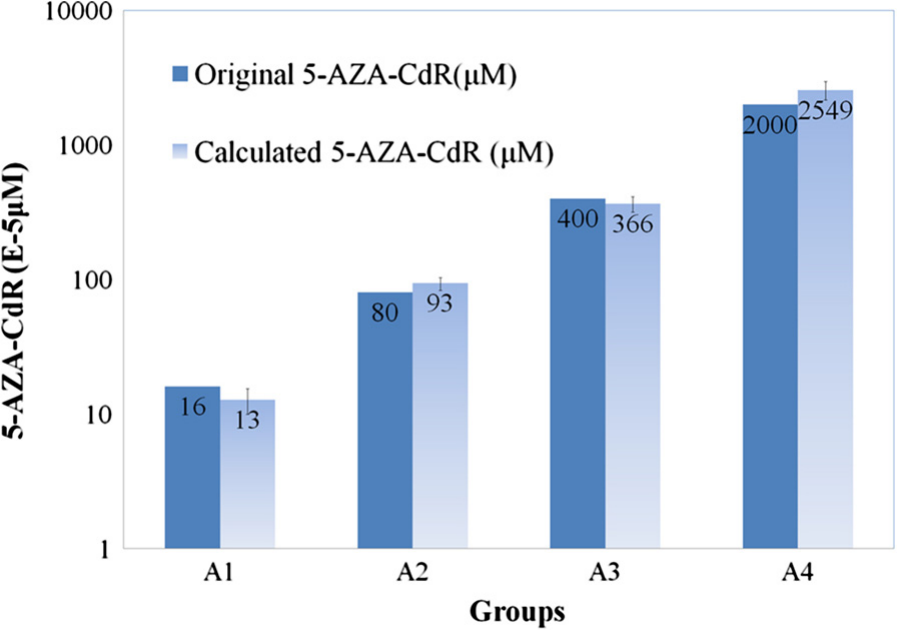
Four samples with different concentrations of 5-AZA-CdR were quantified with TDQ five times. The highest recovery rate (127.5 %) and deviation (13.2 %) were observed for sample with a concentration of 0.02 μM, while the lowest recovery rate (79.5 %) and deviation (11.0 %) were observed for sample with a concentration of 0.00016 μM. The blue bars represent the actual concentration for samples and the gradient bars represent the concentration values calculated from TDQ test

To detect the accuracy and precision of the TDQ assay, five samples with moderate concentrations of 5-AZA-CdR (0.0000, 0.00016, 0.0008, 0.004, 0.02 μM) were applied simultaneously to perform the demethylation quantification five times (Table 2, Fig. 5). Results showed that the deviation results from the repetitive analysis of the four samples (0.00016 ~ 0.02 μM) varied from 11.0 % to 15.7 %. The recovery rate for TDQ test varied from 79.5 % to 127.5 %. The standard line applied here is y = exp (−0.071x^2^ + 2.445x – 19.90) (R^2^ = 0.999).

**Table 2:** Repeated TDQ experiment with cells treated with different doses of 5-AZA-CdR

Groups	Original 5-AZA-CdR (μM)	n	PFC (%)	RSD (%)	Calculated 5-AZA-CdR (μM)	RR (%)
A0	0.00000	5	2.20 ± 0.46	21.1	-	-
A1	0.00016	5	7.48 ± 0.81	10.8	0.000127	79.5
A2	0.00080	5	8.72 ± 1.09	12.5	0.000933	116.7
A3	0.00400	5	9.66 ± 1.51	15.7	0.003655	91.4
A4	0.02000	5	11.18 ± 1.47	13.1	0.025493	127.5

Note. PFC is the percentage of positive fluorescence cells of the total cells. RSD is the relative standard deviation. RR is the Recovery Rate of the 5-AZA-CdR calculated for the original 5-AZA-CdR. There are significant differences for ANOVA analysis of PFC between all groups barring groups A1 and A2 and groups A2 and A3

### Optimal check time for TDQ test

The optimal check time for TDQ test is the suitable time for the user to collect cells for quantification of fluorescence intensity with flow cytometry. The fluorescence intensity of the cells may change at different time of the TDQ timeline. To explore the optimal check time, we took a series of microscope images every 6 h when the 5-AZA-CdR samples were pipetted into the cell culture medium. Images with meaningful fluorescence were found beginning at Hour 36, and ascended to the peak plat period with the brightest fluorescence between Hour 54 and Hour 72. Additional TDQ test of 5-AZA-CdR treatment with different dosages (0.00016, 0.00080 and 0.00400 μM) was conducted (Additional file 3: Table S3). Therefore, Hour 60 was selected as the optimal check time for the entire TDQ test duration.

### Quantification of demethylation potential by TDQ in aquatic samples

To explore the applicability of TDQ method for the quantification of the demethylation of actual aquatic samples, 20 aquatic samples from China’s Bohai Bay were collected to prepare the inorganic extracts for further experimentation. Positive demethylation potentials ranging from 0.00004 to 0.20053 μM 5-AZA-CdR toxicity equivalents can be observed in eight samples. A sample (MS06) extract from Beitangkou showed the highest demethylation toxic equivalency (DTE) at 0.20053 μM 5-AZA toxicity equivalent (Table 3). Cd and As were found at 34.73 mg/Kg and 1.12 mg/Kg in this sample extract with the later ICP-Mass analysis; both concentrations are higher than the maximum allowable levels for Cd and As in aquatic food materials in China. All of the samples with observable demethylation potential included the *Silurus asotus Linnaeus, Sinonovacula constricta, Bullacta exarata, Venerupis variegata, Scapharca subcrenata,* and *Rapana venosa.*

**Table 3:** The demethylation potential and the pollutants of inorganic extracts of aquatic samples from Bohai bay

Samples	Sites	Name	PFC (%)	5-AZA-CdR DTE (μM)	Cd	As	Cr	Mn	Ni	Pb	Hg
MS01	A	*Rapana venosa*	6.26	0.00000	0.49	2.57	0.65	10.70	0.46	0.52	0.21
MS02	A	*Argopectens irradias*	2.41	0.00000	0.12	0.32	0.63	2.99	0.21	0.43	0.13
MS03	A	*Silurus asotus Linnaeus*	2.45	0.00000	0.59	0.98	0.46	13.10	0.38	0.16	0.09
MS04	A	*Sinonovacula constricta*	0.78	0.00000	0.38	0.31	0.97	3.78	0.53	0.28	0.10
MS05	B	*Sinonovacula constricta*	12.52	0.09611	7.38	0.35	0.35	14.28	1.36	0.90	0.02
MS06	B	*Silurus asotus Linnaeus*	13.30	0.20053	34.73	1.12	0.42	13.21	0.34	0.18	0.07
MS07	B	*Scapharca subcrenata*	8.60	0.00066	4.66	0.26	0.22	18.00	0.81	0.15	0.01
MS08	B	*Venerupis variegata*	2.37	0.00000	0.51	1.43	2.17	6.22	2.13	0.21	0.02
MS09	C	*Bullacta exarata*	4.93	0.00000	0.85	1.99	0.43	3.84	0.49	0.11	0.08
MS10	C	*Sinonovacula constricta*	2.41	0.00000	0.27	0.24	0.24	13.87	1.27	0.86	0.00
MS11	C	*Venerupis variegata*	2.38	0.00000	0.46	1.36	1.98	5.96	2.01	0.13	0.01
MS12	C	*Venerupis variegata*	10.17	0.00628	5.35	1.68	2.42	6.68	2.39	0.25	0.02
MS13	D	*Venerupis variegata*	3.95	0.00000	4.99	1.49	2.33	6.53	2.25	0.24	0.03
MS14	D	*Sinonovacula constricta*	7.01	0.00005	2.31	0.28	0.28	14.03	1.32	0.96	0.00
MS15	D	*Rapana venosa*	6.95	0.00004	0.58	0.94	0.67	16.10	0.55	0.19	0.04
MS16	D	*Venerupis variegata*	3.13	0.00000	7.11	0.68	0.94	32.74	0.63	0.41	0.05
MS17	E	*Venerupis variegata*	3.91	0.00000	0.45	1.33	2.23	6.12	2.11	0.17	0.03
MS18	E	*Scapharca subcrenata*	7.04	0.00005	5.04	0.23	0.41	8.01	0.89	0.29	0.03
MS19	E	*Bullacta exarata*	10.95	0.01701	6.85	0.33	0.71	12.05	0.32	0.46	0.05
MS20	E	*Oratosquillina interrupta*	2.35	0.00000	0.63	0.37	0.45	4.67	1.63	0.21	0.06
FSV (mg/Kg)					2.0	0.5	2.0	-	-	1.0	0.5

Note. Sampling Sites: A, Caijiabao; B, Beitangkou; C, Gaoshaling; D, Nanpaihe; E, Yugangkou. PFC is the percentage of positive fluorescence cells of the total cells. 5-AZA-CdR DTE: 5-AZA-CdR toxicity equivalent for demethylation potential (μM). All of the elements are the ICP-MS results for wet weight (mg/Kg). FSV is the standard value of the maximum levels for certain contaminants in marine fishes and crustaceans in China (GB 2762–2012). The FSV of Hg is replaced by those of methyl mercury. – The standard values of Mn and Ni are absent in this national food safety standard

The samples (MS06 and MS05) of *Silurus asotus Linnaeus* and *Sinonovacula constricta* from Beitangkou showed the worst DTE. According to the ICP-MS results for all eight samples, the abnormal rates of Cd, As, and Cr were 87.5 %, 37.5 %, and 12.5 % with the maximum levels for certain food contaminants in China (GB 2762–2012), respectively.

## Discussion

This study describes a method for the quantification of the total DNA demethylation potential of aquatic food samples within one week based on artificial methylation and the useful reporter gene. Currently, the Bohai Bay area is ranked as one of the most heavily polluted coastal areas in China [17, 18]. It is therefore reasonable to explore the novel demethylation potential of the aquatic materials in this area for food safety and additional dietary health risk assessment.

One precondition of this study is the hypothesis that demethylation potential is possibly an innate part of the comprehensive negative effect of environmental pollution, with the counterpart of mutation and chromosome aberration [19]. Dozens of chemical pollutants revealed obvious effects on the steady methylation balance of genomic DNA. DNA methylation changes in whole blood are associated with exposure to environmental contaminants such as cadmium, mercury, lead, and bisphenol A [20], implying that the demethylation potential of the aquatic samples from heavily polluted marine areas should be a focus of health risk assessments of the local residents. Chronic exposure to arsenic leads to global DNA demethylation and aberrant gene expression [21–24], and even short-term exposure to arsenic can disturb DNA methylation patterns [25]. Environmental cadmium exposure is associated with DNA hypomethylation in peripheral blood [5, 26], and some aquatic samples extract with higher concentration of Cd also reveal obvious positive demethylation potential after the TDQ test.

The method of evaluating the demethylation potential of aquatic food samples is meaningful and few studies have reported on it thus far. A detection system for demethylating agents using an endogenous promoter CpG island was reported [10], but fluorescence cannot be quantified so precisely using only imaging and the results may overlook the confounding cytochromes P450 activation effect of environmental pollutants in hepatic cells. Moreover, cell lines using an endogenous, epigenetically silenced reporter are convenient for screening demethylating agents [27], but the methylation may be spontaneously lost after many generations and H1299 lung cancer cells are not the most suitable cell candidates due to the shortage of cytochromes P450 enzymes.

The TDQ method has several advantages in case of quantifying the total demethylation potential of food samples. First, the task of assessing the hazards of such complex mixtures is complicated, because the actual composition of these mixtures is rarely known and the behavior of mixture components is not fully understood [28]. To provide more information for food safety, the TDQ method can screen the samples with greater demethylation potential and likely samples may be selected for further scanning analysis of concrete pollutants. Second, the method can give an accurate relative quantification of the total demethylation potential of the aquatic sample extracts with 5-AZA-CdR DTE, which is more convenient for the direct comparison of health risks for different food samples. Third, comparing to cells from other organs, the Hep G2 cell line is more suitable for toxicity evaluation of pollutants and will help the TDQ test results more reliable. Not only are the hepatic cells just the target cells for toxicity test, but also the hepatic cells may help active pollutants possibly with cytochromes P450 enzymes as well [29, 30]. Moreover, the TDQ method is quick and easy to conduct using common equipments.

Despite the advantages above, compared with the endogenous promoter, a main drawback of TDQ is the exogenous CMV promoter used in this method. There are possibly some pollutants may have demethylation potential and not be sensitive to the demethylation of exogenous promoter, therefore false negative results for the TDQ test might existed. Strong fluorescence may result first from total demethylation potential and the possible later activation of transcription factors, meaning that false positive cases may appear in the results. The possibility of false positives is small, however, due to the rarity of samples with positive demethylation potential and strong activation of transcription factors. Therefore, the explaining the negative results of aquatic samples extract for TDQ test should be cautious. The TDQ test provides a primary evaluation of total demethylation potential, but the possible mechanisms underlying positive samples should be clarified with further research.

## Conclusions

In summary, the TDQ is a meaningful and rapid assay for the relative quantification of the DNA demethylation potential of aquatic food extracts and is a candidate method for the total toxicity evaluation system for the health risk assessment of dietary environmental exposure with respect to epigenetic mechanisms.

## Methods

### TDQ principle

The paper presents a transient test for the demethylation potential of environmental samples. Hep G2 Cells are transfected transiently with a plasmid encoding fluorescent protein and with a highly methylated promoter. Demethylation can then be evaluated directly from an increase in fluorescence using flow cytometry.

The principle of the TDQ is as follows. First, the CMV promoters of the enhanced green fluorescence gene in pEGFP-C3 plasmid were heavily methylated artificially *in vitro* and the plasmids were transfected into Hep G2 cell lines using the FuGENE HD transfection reagent. Then, the extracts from different target seafood samples were incorporated into the common Hep G2 cell culture medium for simultaneous co-incubation. If the extract of aquatic sample A has strong total demethylation potential for the promoter, the methylation level will decrease and more GFP protein will be expressed, which will be shown by the observation of a strong green fluorescence of the experimental Hep G2 cells under microscopy. If the extract of aquatic sample B has weak demethylation potential, the methylation level will be almost unchanged and very little green fluorescence protein will be expressed, resulting in the observation of a light green fluorescence. Simultaneously using a standard curve of the positive demethylation agent 5-AZA-CdR from the experiment, the total demethylation potential, termed the 5-AZA-CdR demethylation toxic equivalency (DTE), of all the extracts from the target aquatic samples can be quantified within one week (Fig. 1).

The total time for the TDQ test, not including the time for some other preparation work, is less than 70 h. For The TDQ test of aquatic samples extract, there are some preparation should be completed before the initiation step of plasmid transfection. The pEGFP-C3 plasmid should be methylated in advance or stored in −80 °C. The Hep G2 cells should be cultured in good performance for the later transient transfection. Once the TDQ test initiates, the whole experiment can’t be paused until the terminal of the cells harvest. At the initiate Hour 0, the cells should keep in good living performance; 24 h later, the cells are transfected with methylated pEGFP-C3 plasmid. At Hour 30, the cells are co-incubated with the 5-AZA-CdR or samples extract for demethylating test. At Hour 60, all the cells from different cell groups are harvested for fluorescence intensity test with flow cytometry at the same check time.

Due to possible high concentration of pollutants in some aquatic samples extract and their possible toxicity to the Hep G2 cells, it is necessary to find a proper volume range of each extract for TDQ test. Before the initiation of TDQ test for aquatic samples, cellular toxicity of all samples extract should be assessed with MTS cellular viability assay in advance and a proper volume range of each extract added into the cell culture medium for TDQ test may be decided.

### Artificial methylation of pEGFP-C3 plasmid

A large quantity of the commercial C3 plasmid was prepared with transformation of E. coli in advance. Then the methylated pEGFP-C3 plasmid with enough volume (more than 500 mg) should be prepared and stored in −80 °C after purification for later TDQ experiment. The total amount of methylated pEGFP-C3 plasmid for TDQ test of 10 aquatic samples extract should be no less than 1 mg. The volume of plasmid for a single methylation reaction system one time is about 500 ng according to the instruction. The unmethylated plasmid DNA was digested with the restriction enzymes MspΙ and HpaΙΙ, and then the short DNA fragments containing the CMV promoter (S) and the complementary long DNA fragments (L) were recovered. Fragment S was methylated completely with M.SssI methyltransferase (New England Biolabs, MA, USA) according to the instructions. The methylated fragment S was then connected back to the unmethylated fragment L for a complete PEGFP-C3 Plasmid DNA cycle with T4 DNA ligase (New England Biolabs, MA, USA) by incubating overnight at 16 °C. The methylated DNA cycle was purified with Plasmid DNA Purification System (Promega, WI, USA).

### Cell culture and MTS cellular viability assay

According to the timeline of the TDQ test, Hour 0 is the initial time for test of aquatic samples. If you are planning to conduct the TDQ test for some samples, the cells should be cultured in good performance for the later transient transfection at Hour 0. The human liver cancer cell line Hep G2 from the China Type Culture Collection (Chinese Academy of Medical Sciences, Beijing, China) grows in DMEM supplemented with 1 % NEAA (Chinese Academy of Medical Sciences, Beijing, China) and 10 % fetal bovine serum (JRH Bioscience, TX, USA) in 37 °C. Once the cells reached 90 % confluence, cellular viability was measured using the -(4,5-dimethylthiazol-2-yl)-5-(3- carboxymethoxyphenyl)-2-(4-sulfophenyl)-2H-tetrazolium (MTS) Cell Viability Assay (Promega Corporation, WI, USA) according to the manufacturer’s instructions.

### Transfection of methylated pEGFP-C3 plasmid

The good living performance of Hep G2 cells should be kept for at least 24 h. Then the pEGFP-C3 plasmid with the fully methylated CMV promoter was transfected into the Hep G2 cells with the FuGENE HD transfection reagent (F. Hoffmann-La Roche Ltd, Basel, Switzerland) at Hour 24. Cells were seeded for a normal incubation condition at a density of 3 × 10^5^ cells/10-cm dish. Approximately 2 μg methylated pEGFP-C3 plasmid DNA was mixed with 100 ml opti-MEM medium according to the instruction manual. This reagent was carefully pipetted into the medium containing the diluted pEGFP-C3 DNA (0.02 μg/μl). The transfection reagent and plasmid complex was incubated for 15 min at room temperature in advance, after which the complex was added to the cells in a drop-wise manner. The wells were swirled carefully to ensure distribution over the entire plate surface.

To keep efficiency and economy for the pEGFP-C3 plasmid transfection into the Hep G2 cells, it is meaningful to find the right and suitable volume of transfection reagent usage for common plasmid and cells density. After screening for the suitable volume (3, 4, 5, 6, or 7 μl) of the transfection reagent from a pre-experiment of unmethylated plasmids, it was found that Hep G2 cells grew best and their fluorescence was brightest using 7 μl of the transfection reagent. The percentages of positive fluorescence cells for transfection using 7 μl of the transfection reagent are found between 31-52 %, which implies the transformation efficiency is acceptable.

### Cell treatment with 5-AZA-CdR and the aquatic sample extracts

At Hour 30, the cells were treated with different concentrations of 5-AZA-CdR (Sigma, MO, USA), a positive demethylation agent for myelodysplastic syndrome therapy [31], and the target extract of the aquatic samples which was freshly dissolved in PBS and filtered through a 0.2-μm membrane. At Hour 60, cells were harvested for different purposes, such as DNA methylation quantification of the CMV promoter, real-time polymerase chain reaction (PCR) of EGFP gene expression, fluorescent imaging, and the fluorescence intensity test with simultaneous flow cytometry.

### Methylation quantification of the CMV promoter

The promoter methylation of the C3 plasmid inside the Hep G2 cells was quantified using bisulfite sequencing PCR (BSP), as reported previously [32]. There are 72 CpG sites located in the promoter region and the first part of the EGFP gene code region between 1 and 1046 bp. The total methylation level of 25 CpG sites between 447 and 747 bp were investigated by bisulfite sequencing. In brief, all DNA, including the C3 plasmid within the cells, was collected by serial extraction with phenol/chloroform and ethanol precipitation as usual and was treated with bisulfite sodium. The PCR amplification product of the target CMV promoter region with methylation-specific primers was purified and linked with the pGEM-T vector (Promega, WI, USA) for transfection into the competent bacteria *E. coli* strain DH5α, and later blue-white screening. All of the positive clones were screened for further sequencing with universal primer T7 and SP6 at the Beijing Genomics Institute. The self-designed primers for BSP in the promoter were as follows: forward primer (23 nt) at the site of 447–469 bp with the sequence 5'TAATGGGAGTTTGTTTTGGTATT3', and reverse primer (25 nt) at the site of 1022–1046 bp with the sequence 5'TTATACTCCAACTTATACCCCAAAA3'.

### Relative quantification of EGFP mRNA with real-time PCR

The method of relative quantification of EGFP mRNA via real-time PCR is as previously reported [33]. In brief, total RNA was isolated from cells with Trizol reagent (Invitrogen, AL, USA). First strand cDNA was synthesized using the PrimeScriptTM RT Reagent Kit (Takara, Dalian, China) from an equal amount of RNA. Quantitative PCR was performed with stratagene MX3100 QPCR (Bio-Rad Laboratories, CA, USA) using SYBR Premix Ex TaqTM (Takara, Dalian, China). Samples were analyzed in triplicate. Gene expression values were calculated based on the comparative quantitative method and normalized to values obtained from the amplification of the endogenous control glyceraldehyde-3-phosphate dehydrogenase (GAPDH). The △Ct values for all genes were determined relative to GAPDH. The △△Ct values were calculated using the treated group means relative to the control group means. The fold change data were calculated from the △△Ct values. Quantitative PCR amplification was carried out with the following sets of primers. Primers sequence for the EGFP gene: 5'- TAATGGGAGTTTGTTTTGGTATT-3' (sense), 5'-TTATACTCCAACTTATACCCCAAA A-3' (antisense); primers sequence for the GAPDH gene: 3'-AGGTGAAGGTCGGAGTCAACG-3' (sense), 5' -AGGGGTCATTGATGGCAACA-3' (antisense). For both genes, PCR was performed for 40 cycles at an annealing temperature of 60 °C.

### Fluorescence intensity detection of cells by flow cytometry

Fluorescence intensity was measured first with imaging and then more accurately with flow cytometry. First, cells were washed with PBS and fixed with 4 % paraformaldehyde. Fluorescence was observed using an Olympus IX71 microscope (Olympus, Tokyo, Japan), and images were analyzed with Image Pro Plus 6.0 software for Windows (Media Cybernetics, USA). Cell fluorescence was then assayed using flow cytometry (Altra, Beckman Coulter, USA). One ml of PBS puff was added uniformly to the 24-well plates for cell detachment, after which the cells was transferred into a 1.5-ml tube to remove the supernatant after centrifugation at 2000 g for 3 min. All of the cells in the stream-specific tube were pumped into the flow cytometry to detect the percentage of cells with positive EGFP fluorescence.

### Sampling sites around Bohai Bay and aquatic sampling

The environmental monitoring report shows that Haihe River has been awarded the worst possible water quality rating for 20 years. The aquatic sampling sites were located around the estuary of the Haihe River in the Bohai Bay area. Approximately 20 km separated each of the five sampling sites (Additional file 4: Figure S1), and sampling was carried out from September to October of 2011. The collected mollusks were depurated in filtered seawater for approximately 24 h before being transported to the laboratory with ice freezing. The mollusks’ edible soft tissues were excised by stainless steel scalpels and then thoroughly rinsed with MQ water to remove extraneous impurities. After sufficient homogenization by a blender, the samples were kept at −20 °C until analysis. Different species of mollusks were identified according to the catalog of marine mollusks in reference books.

### Pretreatment of aquatic samples for TDQ test and ICP-MS analysis

Approximately 50 g (wet weight) of aquatic soft tissues were divided into five parts and each part was weighed in a PTFE digestion container. Fifty ml of concentrated nitric acid was added to each part and left to predigest overnight at 40 °C. Twenty ml of 30 % hydrogen peroxide was then added after cooling. Thereafter the container was covered and placed in a high-pressure stainless steel bomb in an oven. The oven temperature was increased to 160 °C and maintained for 4 h. After cooling, the total solution of five parts was collected to dry and then diluted with 5 ml Milli-Q water (5 g/ml, 10×).

Four ml of aquatic extraction solution was centrifuged with 5000 g for 10 min and the aqueous supernatant was kept sterile after filtration for the next TDQ test using 0.22 μm microfiltration membranes. The other 1 ml solution was transferred to a PET bottle and stored at −20 °C for later analysis with ICP-MS (Cd, As, Cr, Mn, Ni, Pb, and Hg). All elements were consistent with the certified values, with the recoveries ranging from 81.62 % to 118.70 %. The detection limits of Cd, As, Cr, Mn, Ni, Pb and Hg were 5.0, 5.0, 30, 60.00, 5.0, 10.0, and 5.0 μg/Kg, respectively.

### Demethylation potential quantification of aquatic samples with the TDQ test

For aquatic samples extract, if the concentrations of some pollutants contained are very high, the extract might be toxic to the Hep G2 cells. Therefore, it is necessary for us to find a proper volume range of each extract for TDQ test. With an MTS assay for each sample in advance, the proper volume range of each target sample may be found for TDQ test usually. Using the same method as 5-AZA-CdR, the extraction solution of each aquatic sample was added to the different wells at a concentration of 1× in 24-well plate at Hour 30. There were three parallels for each sample. The fluorescence intensity of each well was measured first with imaging and then with flow cytometry analysis at Hour 60.

### Statistical analysis

The total demethylation potential of an aquatic sample is expressed by the DTE of 5-AZA-CdR after the TDQ test. All statistical analysis was performed using SPSS16.0 for Windows. Linear regressions and Pearson correlation was conducted to evaluate the association between the concentrations of 5-AZA-CdR and the methylation levels of the CMV promoter, the relative levels of EGFP mRNA and fluorescence intensity. Data were expressed as the mean ± SD. Differences with a P value of less than 0.05 were considered to be statistically significant, ANOVA was for difference analysis of groups.

## Additional files

Additional file 1: Table S1. Quantitative real-time PCR of the EGFP mRNA for 5-AZA-CdR treated Hep G2 cell lines. (PDF 114 kb) Additional file 2: Table S2. The detection limit of TDQ experiment with repetitive tests of 5-AZA-CdR. (PDF 11 kb) Additional file 3: Table S3. The optimal check time for TDQ test with repetitive tests of 5-AZA-CdR treatment. (PDF 11 kb) Additional file 4: Figure S1. Aquatic sampling sites around the Haihe river estuary of Bohai Sea. All five sites are 15 km from the coastal line and 20 km apart. (PDF 755 kb)

## Abbreviations

5-AZA-CdR: 5-Aza-2'-deoxycytidine
TDQ: quantification method of total demethylation potential
DTE: demethylation toxic equivalency
PFC: percentage of positive fluorescence cells
