# Supplementary material for: A novel quantification method for the total demethylation potential of aquatic sample extracts from Bohai Bay using the EGFP reporter gene
Source: BMC Biotechnol. 2015 Nov 26;15:107. doi: 10.1186/s12896-015-0224-y (PMC4660669; doi:10.1186/s12896-015-0224-y)
Supplement: Additional file 1: Table S1. — Quantitative real-time PCR of the EGFP mRNA for 5-AZA-CdR treated Hep G2 cell lines. (PDF 114 kb) [file 12896_2015_224_MOESM1_ESM.pdf]

Additional file 2

Table 1 Quantitative real-time PCR of the EGFP mRNA for 5-AZA-CdR treated Hep G2 cell lines.

Groups	5-AZA-CdR (μM)	n	EGFP (Ct)	GADPH (Ct)	△CT	△△CT	Relative fold
A0	0.00000	5	15.9	20.87	-4.970	0.000	1.000
A1	0.00016	5	14.16	19.85	-5.690	-0.720	1.647
A2	0.00080	5	21.87	27.66	-5.790	-0.820	1.765
A3	0.00400	5	16.88	22.79	-5.910	-0.940	1.919
A4	0.02000	5	18.95	27.54	-8.590	-3.620	12.295

Note. △Ct is equal to EGFP (Ct) minus GADPH (Ct) with the same concentrations of 5-AZA-CdR (μM). △△Ct is equal to= the △Ct of different concentrations of 5-AZA-CdR (μM) minus the △Ct of the control concentration. Relative fold is calculated using the equation Relative fold = exp(2, -△△Ct). There are significant differences for the ANOVA analysis of EGFP mRNA levels between all groups barring groups A1 and A2, groups A2 and A3, and groups A1 and A3.
