# Supplementary material for: A novel quantification method for the total demethylation potential of aquatic sample extracts from Bohai Bay using the EGFP reporter gene
Source: BMC Biotechnol. 2015 Nov 26;15:107. doi: 10.1186/s12896-015-0224-y (PMC4660669; doi:10.1186/s12896-015-0224-y)
Supplement: Additional file 2: Table S2. — The detection limit of TDQ experiment with repetitive tests of 5-AZA-CdR. (PDF 11 kb) [file 12896_2015_224_MOESM2_ESM.pdf]

Additional file 3

Table 2 The detection limit of TDQ experiment with repetitive tests of 5-AZA-CdR.

Groups	n	5-AZA-CdR (μM)	PFC (%)
A0	8	0.0000000	2.59±1.49
A1	8	0.0000025	2.84±1.45
A2	8	0.0000100	3.79±1.72
A3	8	0.0000400	5.61±1.24
A4	8	0.0001600	6.99±1.05
A5	8	0.0006400	8.10±1.02

Note. PFC is the percentage of positive fluorescence cells of the total cells. There are significant differences for ANOVA analysis of the PFCs between group A3 and all the other groups, between group A4 and some other groups (A0, A1, A2, A3), between group A5 and some other groups (A0, A1, A2, A3).
