# Supplementary material for: A novel quantification method for the total demethylation potential of aquatic sample extracts from Bohai Bay using the EGFP reporter gene
Source: BMC Biotechnol. 2015 Nov 26;15:107. doi: 10.1186/s12896-015-0224-y (PMC4660669; doi:10.1186/s12896-015-0224-y)
Supplement: Additional file 3: Table S3. — The optimal check time for TDQ test with repetitive tests of 5-AZA-CdR treatment. (PDF 11 kb) [file 12896_2015_224_MOESM3_ESM.pdf]

Additional file 4

Table 3 The optimal check time for TDQ test with repetitive tests of 5-AZA-CdR treatment.

Groups	Check time (Hrs)	n	PFC-D1 (%)	PFC-D2 (%)	PFC-D3 (%)
T1	36	6	1.38±0.34	1.55±0.34	1.68±0.30
T2	42	6	2.10±0.22	2.48±0.24	2.88±0.16
T3	48	6	4.27±0.46	4.97±0.45	5.97±0.61
T4	54	6	6.48±0.49	7.75±0.44	8.80±0.53
T5	60	6	6.97±0.22	8.03±0.42	9.15±0.42
T6	66	6	7.10±0.58	8.00±0.50	9.18±0.61
T7	72	6	7.47±0.22	8.58±0.47	9.27±0.58

Note. PFC is the percentage of positive fluorescence cells of the total cells. PFC-D1, PFC-D2 and PFC-D3 represent the PFC values for cells treated with different dosages of 5-AZA-CdR (0.00016, 0.00080, 0.00400 μM). There are significant differences for ANOVA analysis of PFC-D1 between all groups for different check time barring groups T5 and T6 and groups T6 and T7. There are significant differences for ANOVA analysis of PFC-D2 between all groups for different check time barring groups T4 and T5, groups T4 and T6 and groups T5 and T6. There are significant differences for ANOVA analysis of PFC-D3 between all groups for different check time barring groups T4 and T5, groups T4 and T6, groups T4 and T7, groups T5 and T6, groups T5 and T7, and groups T6 and T7.
