# Supplementary figures and images for: A novel quantification method for the total demethylation potential of aquatic sample extracts from Bohai Bay using the EGFP reporter gene
Source: BMC Biotechnol. 2015 Nov 26;15:107. doi: 10.1186/s12896-015-0224-y (PMC4660669; doi:10.1186/s12896-015-0224-y)

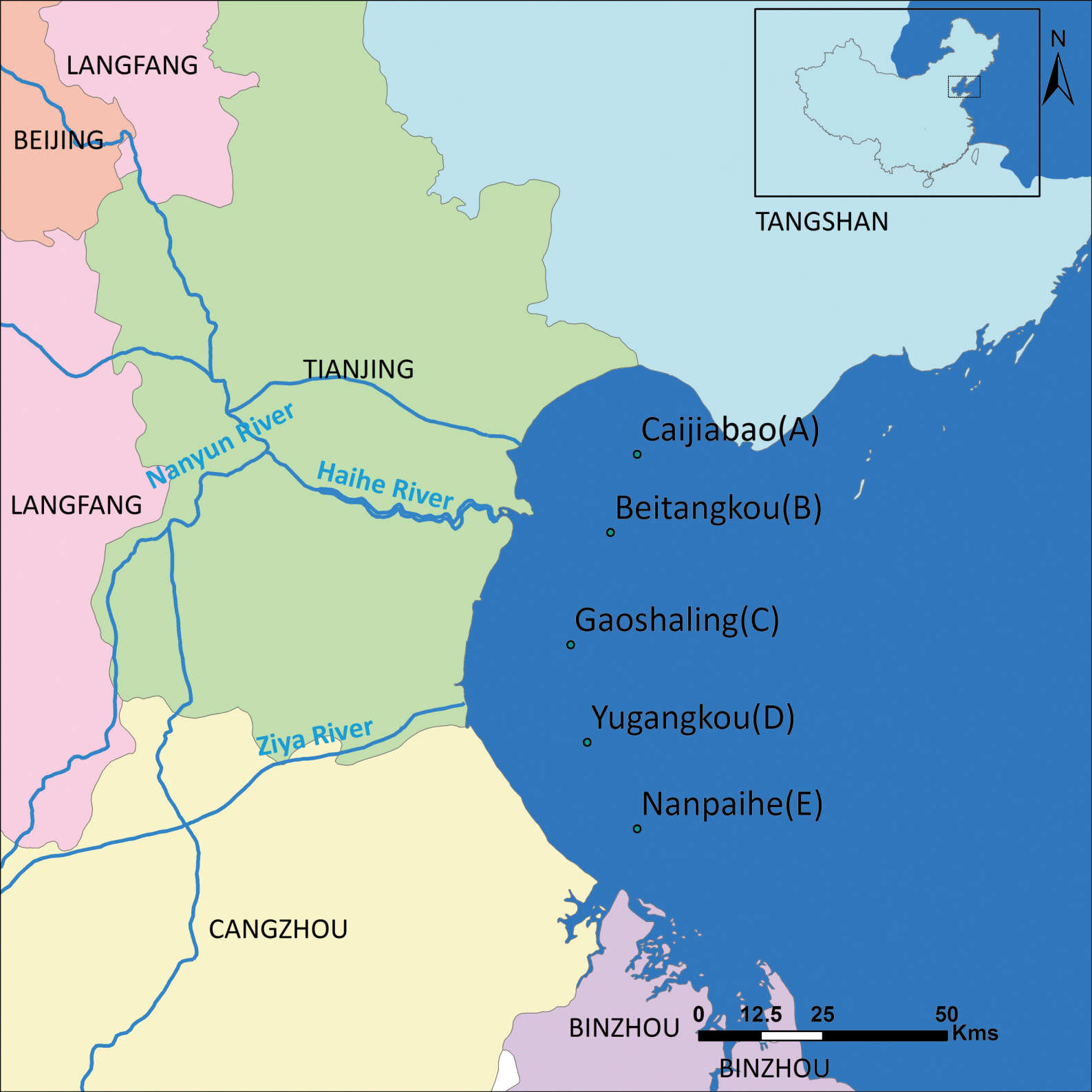

Supplement: Additional file 4: Figure S1. — Aquatic sampling sites around the Haihe river estuary of Bohai Sea. All five sites are 15 km from the coastal line and 20 km apart. (PDF 755 kb) [file 12896_2015_224_MOESM4_ESM.pdf]
